# Supplementary material for: Translation regulatory factor BZW1 regulates preimplantation embryo development and compaction by restricting global non-AUG Initiation
Source: Nat Commun. 2022 Nov 4;13:6621. doi: 10.1038/s41467-022-34427-x (PMC9636173; doi:10.1038/s41467-022-34427-x)
Supplement: Supplementary file 1 — Supplementary Information [file 41467_2022_34427_MOESM1_ESM.pdf]

Supplementary Materials for

Translation Regulatory Factor BZW1 Regulates Preimplantation Embryo Development and Compaction by Restricting Global non-AUG Initiation

Jue Zhang[#], Shuai-Bo Pi[#], Nan Zhang, Jing Guo, Wei Zheng, Lizhi Leng, Ge Lin[*], Heng-Yu Fan[*]

*Corresponding authors:
Prof. Heng-Yu Fan, Life Sciences Institute, Zhejiang University, 866 Yuhangtang Rd., Hangzhou 310058, China. Tel: 86-571-8898 1370; E-mail: hyfan@zju.edu.cn

Dr. Ge Lin, NHC Key Laboratory of Human Stem and Reproductive Engineering, School of Basic Medical Science, Central South University, 410078, Changsha, China. Tel: 86-0731-13187057355; E-mail: linggf@hotmail.com

The PDF file includes:

Supplemental figures and figure legends: Figs. S1 to S6

Supplementary Table 1. Antibody information

Supplementary Table 2. Real time-PCR primers information

Supplementary Table 3. siRNA sense sequence information

Other Supplementary Material for this manuscript includes the following:

Supplementary Data 1. (.xlsx)

Author Checklist (.docx)

Editorial-policy-checklist (.pdf)

Reporting-summary (.pdf)

Source Data file (.xlsx)

Supplementary Materials

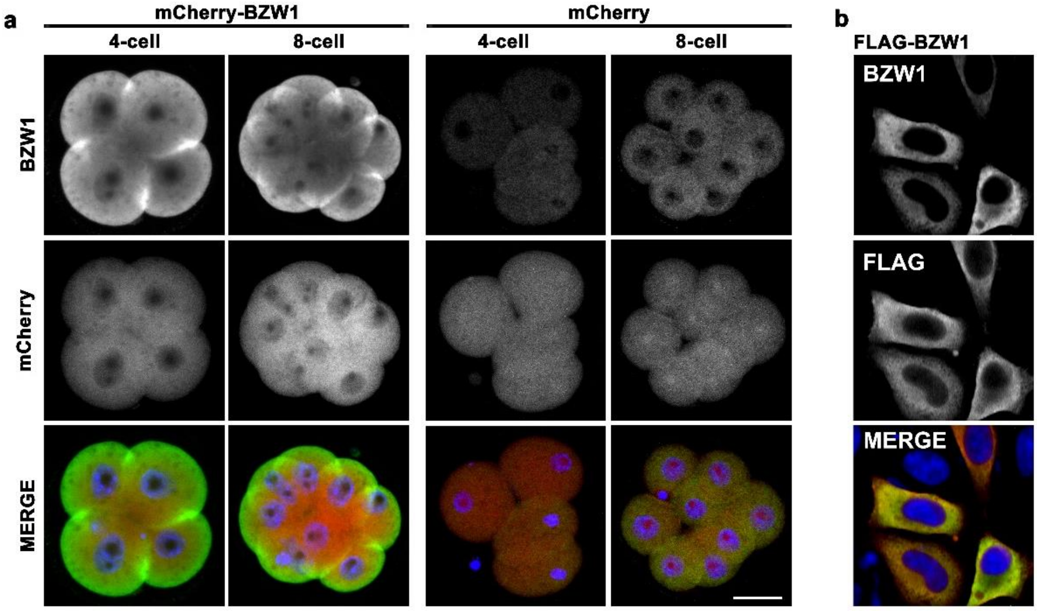

Figure S1. **a:** Confocal microscopic images of BZW1 (green) and mCherry (red) immunofluorescence in preimplantation embryos in 4- and 8-cell stage after mCherry or mCherry-Bzw1 mRNA microinjection in zygotic stage. DNA was counterstained with DAPI (blue). The greyscale pictures were shown for single channel. Scale bar, 25μm. The experiment was repeated more than two times with similar results. **b:** Because of the BZW1 antibody cannot recognize human endogenous BZW1, we expressed the FLAG-BZW1 plasmids into HeLa cells for 24h. The experiment was repeated more than two times with similar results. Immunofluorescence results of FLAG (red) and BZW1 (green) show that BZW1 is a cytoplasmic protein in HeLa cell. Scale bar, 10μm.

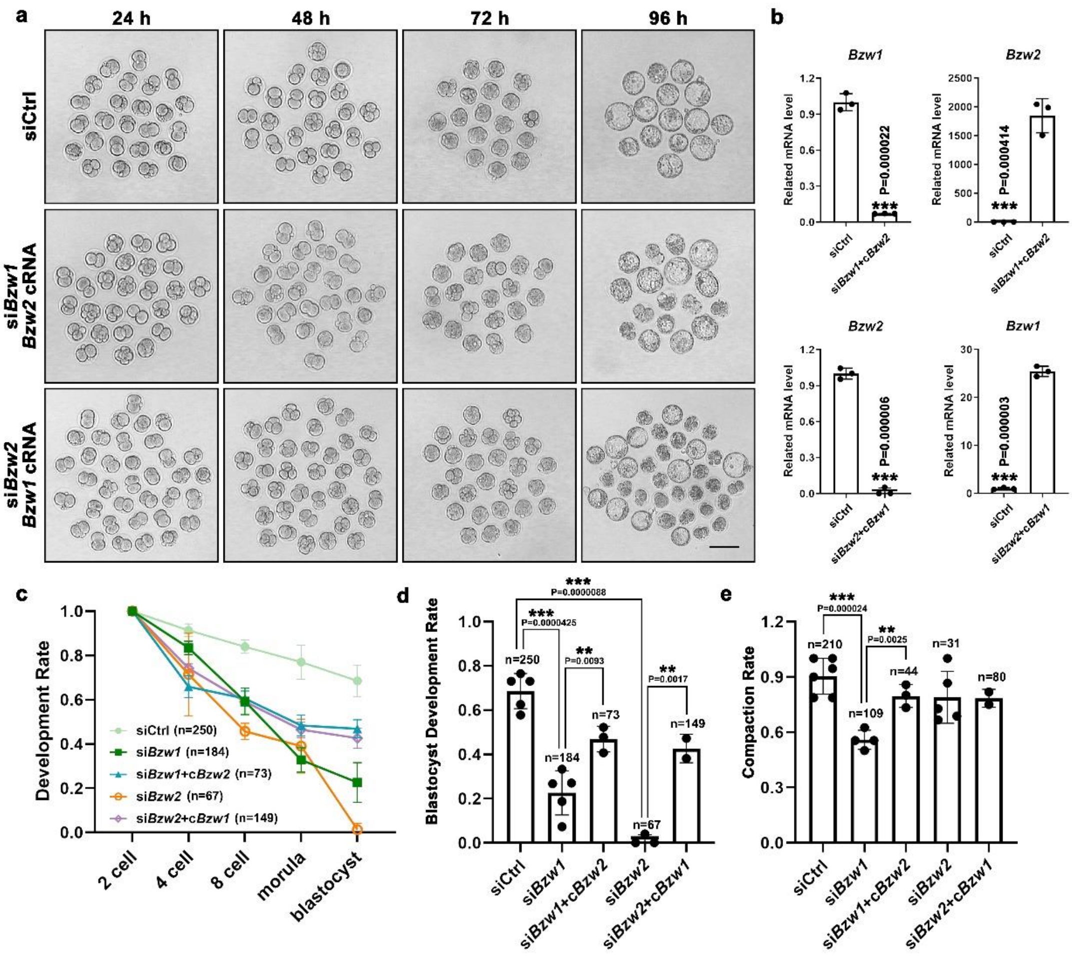

Figure S2. a: Representative embryo images were taken from the embryos at 24, 48, 72, and 96 h after indicated siRNA and mRNA micro-injection in zygotes. Scale bar, 100 μm. **b:** RT-PCR results showing the mRNA levels of *Bzw1* and *Bzw2* in embryo at 2-cell stage after co-microinjection of si*Bzw1* and *Bzw2* mRNA in zygotes. n=5 embryos in every sample. Data are presented as mean values. *Gapdh* is served as an internal control. The relative mRNA level in control is defined as 1.0. Error bars, S.D. **c:** The developmental rates of embryos that reached the 2-cell, 4-cell, 8-cell, morula, and blastocyst stages in indicated groups from (S2a, 2c). n indicates the number of embryos were counted. Data are presented as mean values. Error bars, S.D. **d:** The indicated siRNA and mRNA-microinjected embryos cultured for 4 days *in vitro*. The percentage of blastocyst embryos was calculated and it is presented as the developmental rate. n indicates the number of embryos were counted. Data are presented as mean values. **, P < 0.01, ***, P < 0.001, by two-tailed Student's t-test.

Error bars, S.D. **e:** Embryos microinjected with indicated siRNA and mRNA were cultured for 4 days *in vitro*. The percentage of 8-cell embryos that completed compaction was calculated and it is presented as the compaction rate. n indicates the number of embryos were counted. Data are presented as mean values. **, $P < 0.01$, ***, $P < 0.001$, by two-tailed Student's t-test. Error bars, S.D.

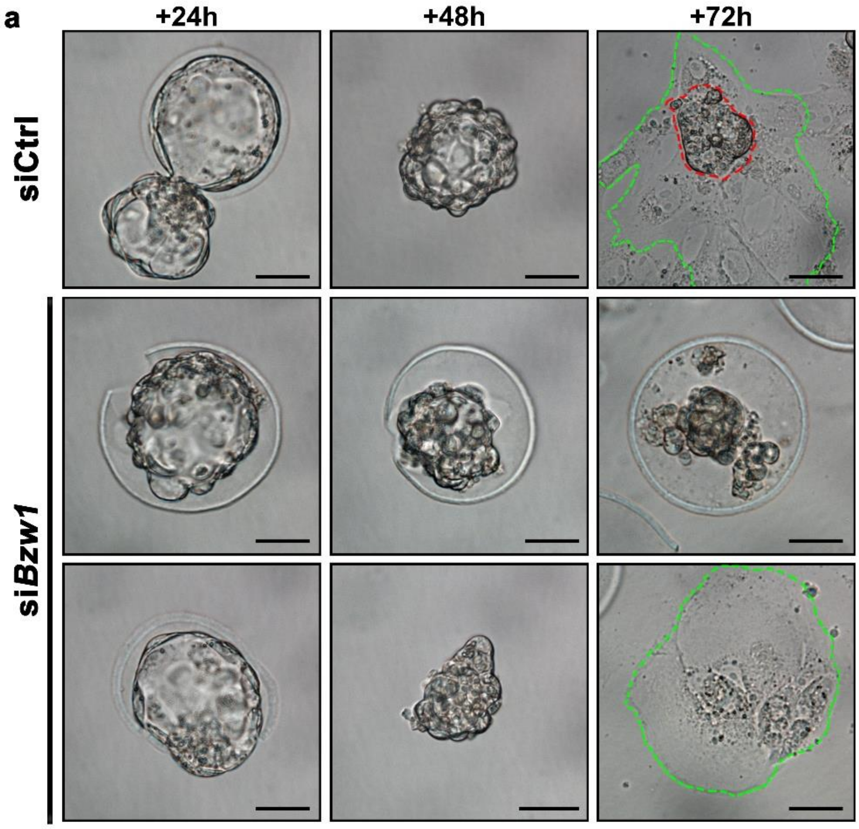

Figure S3. a: Blastocysts are assessed by three-day outgrowth (OG) assay after microinjection of si*Control* or si*Bzw1* in zygotes. DIC images are taken from these blastocysts hatched and attached to the plate after 24 h and 48 h culture, respectively, then formed ICM colony (red line) with proliferating trophoblast cells (green line) by 72 h. At least 30 blastocysts were observed in each experimental group. The experiment was repeated three times with similar results. Scale bars, 25μm.

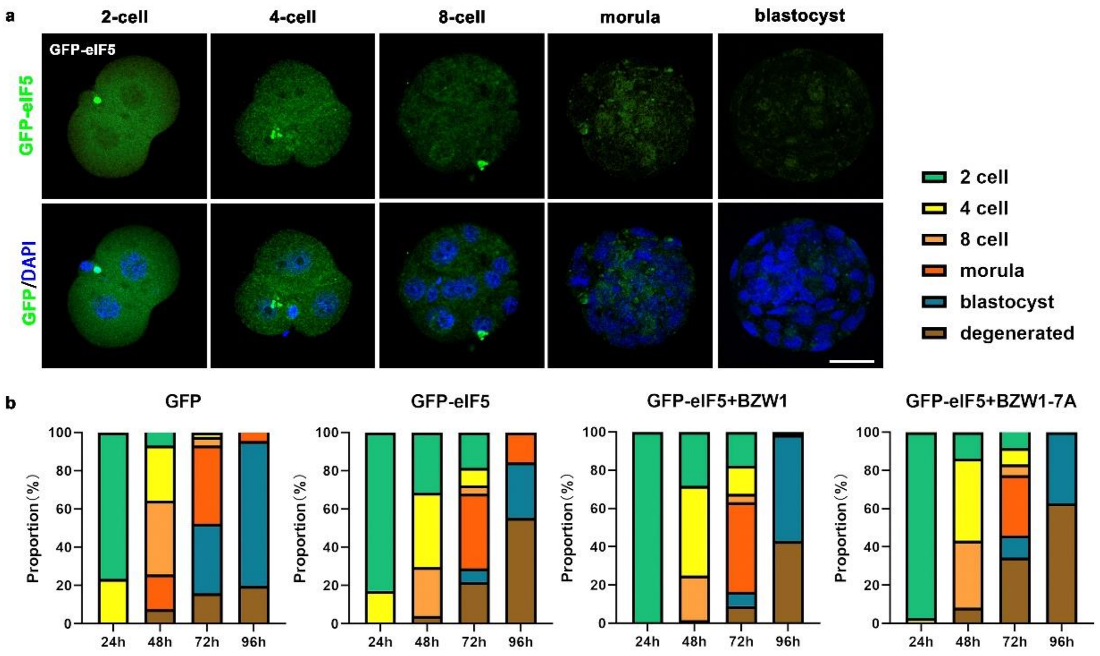

Figure S4. a: Confocal microscopic images of eIF5 (green) immunofluorescence in preimplantation embryos from 2-cell stage to blastocystic stage after GFP-eIF5 mRNA microinjection in zygotic stage. DNA was counterstained with DAPI. Scale bar, 25μm. **b:** Quantification of developmental rates of cultured embryos for every 24h after injection in figure 5d. The embryos were respectively microinjected with mRNAs encoding GFP (as control), GFP-eIF5, GFP-eIF5 and mCherry-BZW1, or GFP-eIF5 and mCherry-BZW1-7A mutants.

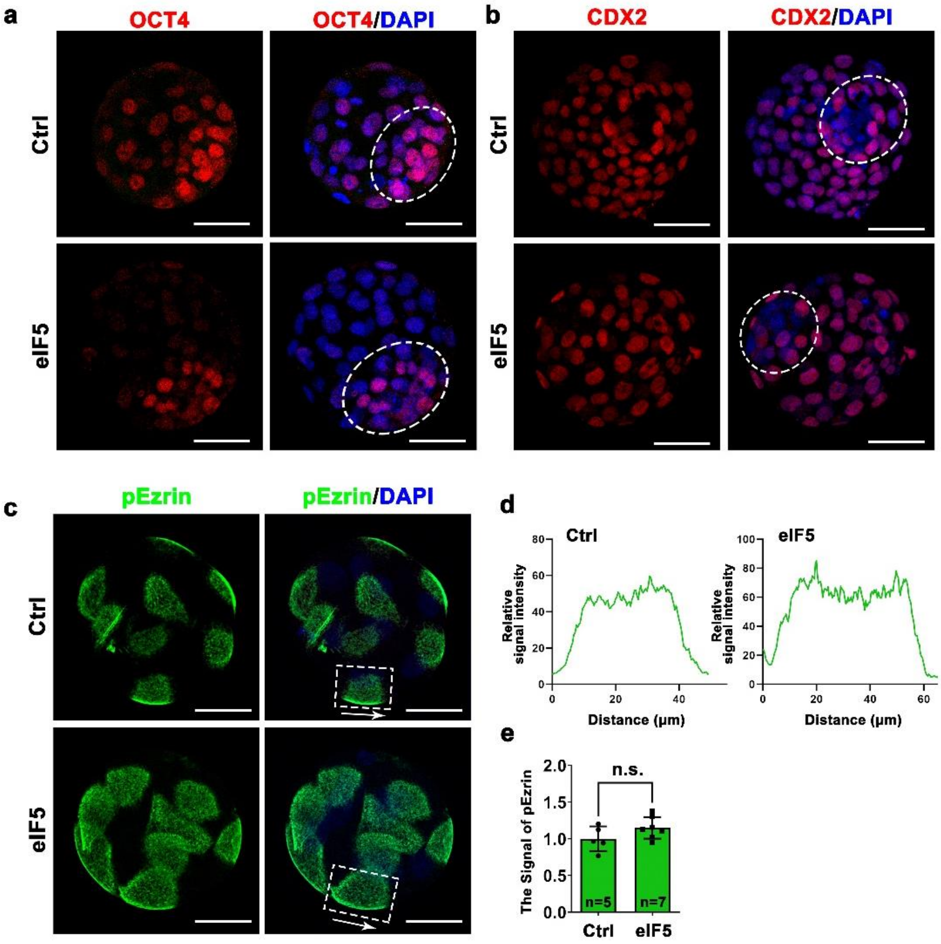

Figure S5. a: Immunofluorescence of OCT4 (red) in Control and over-expressed eIF5 in the embryos develop to blastocyst stage. Scale bar, 25μm. **b:** Immunofluorescence of CDX2 (red) in Control and over-expressed eIF5 in the embryos develop to blastocyst stage. Scale bar, 25μm. **c:** Immunofluorescence of p-Ezrin (green) in Control and over-expressed eIF5 in the embryos fixed when compaction occurs in the control embryos. DNA was counterstained with DAPI. Scale bar, 25μm. At least 15 embryos were observed in each experimental group. White squares labeled denote signal intensity analysis regions in (S5d). Arrow direction was corresponding to the X-axs in (S5d). **d:** Signal intensities of pEzrin^{T567} indicate the level of pEzrin at the cell-contact free surface in (S5c). **e:** Signal intensities of p-Ezrin from (S5c) indicated the level of p-Ezrin in Control and eIF5 over-expressed embryos. n indicates the number of embryos were analyzed. Data are presented as minima, maxima and mean values in the box plots. The relative signal level in control is defined as 1.0. n.s., not significant, by two-tailed Student's *t*-tests. Error bars indicate S.D.

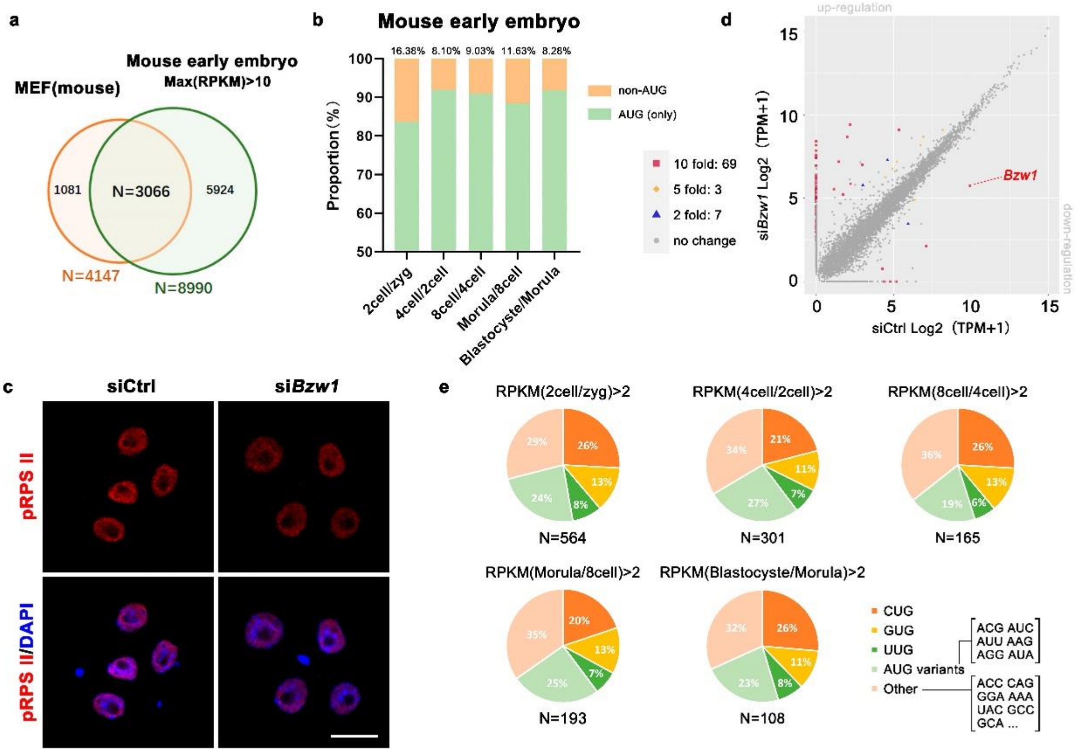

Figure S6. a: Overlap between mRNA expression in mouse early embryo (8990 transcripts, RPKM > 10 in at least one sample for each stage) and transcripts contain at least one upstream TIS (uTIS) used non-AUG as start codon in mouse MEF. **b:** The proportion of non-AUG-initiation mRNA expressed up-regulated in every stage. Genes showed changes of transcripts between two consecutive developmental stages in mouse was more than 2 times were selected. **c:** Confocal microscopic images of p-RPSII (red) immunofluorescence 4-cell embryos after siControl or si*Bzw1* microinjection in zygotes. DNA was counterstained with DAPI (blue). The experiment was repeated two times with similar results. Scale bar, 25μm. **d:** Scatter plots of RNA-seq data illustrated transcriptional change in si*Bzw1* compared with Control. Genes showing an expression difference larger than 2-fold, 5-fold and 10-fold were labeled as blue, yellow and red dots. **e:** Non-AUG start codon composition of uTIS codons identified in every group as S6b. The original dates are from RNA-Seq in mouse early embryo and GTI-seq in mouse MEF.

Supplementary Table 1.

Antibody information

**Antibody name**	**Manufacture (catalogue number)**	**Applications (working dilution)**
anti-BZW1	Proteintech (19917-1-AP)	IF (1:200)<br>WB (1:500)
anti-DDB1	Epitomics (3821-1)	WB (1:5000)
anti- FLAG	Santa Cruz (sc-807)	IF (1:200)
anti-HA tag	CST (#3724)	WB (1:20,00)
anti-pEzrin (Thr567)	CST (#3726)	IF (1:500)
anti-E-Cad	CST (#3195)	IF (1:500)
anti-Nanog	Sigma (SAB5700793)	IF (1:50)
anti-OCT4	Sigma (ZRB1101)	IF (1:200)
anti-CDX2	BioGenex (MU392A-UC)	IF (1:200)
anti-DDB1	Abcam (#3821-1)	WB (1:20,00)
anti-GFP	Abcam (ab32146)	WB (1:500)
Anti-cMyc	Invitrogen (13-2500)	WB (1:300)
anti-Histone H3	CST (#4499s)	WB (1:10,00)

Supplementary Table 2.

Real time-PCR primers information

Name	Species	Sequence
Bzw1-FP	Mouse	AGCAGCAAAAACCAACGCTAT
Bzw1-RP	Mouse	GCCAGCCACCAGAATGTCA
Fgfr1-FP	Mouse	TAATACCACCGACAAGGAAATGG
Fgfr1-RP	Mouse	TGATGGGAGAGTCCGATAGAGT
Fgfr2-FP	Mouse	CCTCGATGTCGTTGAACGGTC
Fgfr2-RP	Mouse	CAGCATCCATCTCCGTCACA
Oct3/4-FP	Mouse	TTGGGCTAGAGAAGGATGTGGTT
Oct3/4-RP	Mouse	GGAAAAGGGACTGAGTAGAGTGTGG
Sox21-FP	Mouse	CCTACTCACTGCTCGACCTG
Sox21-RP	Mouse	TTGCACGGGATCATGTAGCC
Gjb5-FP	Mouse	TGTGGGGAGACGACCAGAA
Gjb5-RP	Mouse	CGGGATTCGGGTAAAGGTAAC
Nanog-FP	Mouse	TTCTTGCTTACAAGGGTCTGC
Nanog-RP	Mouse	AGAGGAAGGGCGAGGAGA
Sox2-FP	Mouse	GCGGAGTGGAAACTTTTGTCC
Sox2-RP	Mouse	CGGGAAGCGTGTACTTATCCTT
Sumo2-FP	Mouse	AAGGAAGGAGTCAAGACTGAGAA
Sumo2-RP	Mouse	CGGAATCTGATCTGCCTCATTG
Dusp6-FP	Mouse	ATAGATACGCTCAGACCCGTG
Dusp6-RP	Mouse	ATCAGCAGAAGCCGTTCGTT

Supplementary Table 3.

siRNA sense sequence information

**Name**	**Species**	**Sequence**
NC	Mouse & Human	5’- UUCUCCGAACGUGUCACGUTT -3’
si*Bzw*1	Mouse	5’- TGAGTCTGAAGCTGAAGAA -3’
si*Bzw*2	Mouse	5’- CATGAAACCATCCGAAACT -3’
