# Supplementary material for: Translation regulatory factor BZW1 regulates preimplantation embryo development and compaction by restricting global non-AUG Initiation
Source: Nat Commun. 2022 Nov 4;13:6621. doi: 10.1038/s41467-022-34427-x (PMC9636173; doi:10.1038/s41467-022-34427-x)
Supplement: Supplementary file 3 — Description of Additional Supplementary Files [file 41467_2022_34427_MOESM3_ESM.pdf]

Description of Additional Supplementary Files

File Name: Supplementary Data 1
Description: TPM of mRNA level in si*Bzw1* vs siCtrl. at 4 cell stage.
