# Supplementary material for: Translation regulatory factor BZW1 regulates preimplantation embryo development and compaction by restricting global non-AUG Initiation
Source: Nat Commun. 2022 Nov 4;13:6621. doi: 10.1038/s41467-022-34427-x (PMC9636173; doi:10.1038/s41467-022-34427-x)
Supplement: Supplementary file 5 — Reporting Summary [file 41467_2022_34427_MOESM5_ESM.pdf]

nature portfolio

Corresponding author(s): Heng-Yu Fan

Last updated by author(s): Oct 5, 2022

Reporting Summary

Nature Portfolio wishes to improve the reproducibility of the work that we publish. This form provides structure for consistency and transparency in reporting. For further information on Nature Portfolio policies, see our Editorial Policies and the Editorial Policy Checklist.

Statistics

For all statistical analyses, confirm that the following items are present in the figure legend, table legend, main text, or Methods section.

n/a	Confirmed	
☐	☒	The exact sample size (*n*) for each experimental group/condition, given as a discrete number and unit of measurement
☐	☒	A statement on whether measurements were taken from distinct samples or whether the same sample was measured repeatedly
☐	☒	The statistical test(s) used AND whether they are one- or two-sided<br>*Only common tests should be described solely by name; describe more complex techniques in the Methods section.*
☐	☒	A description of all covariates tested
☐	☒	A description of any assumptions or corrections, such as tests of normality and adjustment for multiple comparisons
☐	☒	A full description of the statistical parameters including central tendency (e.g. means) or other basic estimates (e.g. regression coefficient) AND variation (e.g. standard deviation) or associated estimates of uncertainty (e.g. confidence intervals)
☐	☒	For null hypothesis testing, the test statistic (e.g. *F*, *t*, *r*) with confidence intervals, effect sizes, degrees of freedom and *P* value noted<br>*Give P values as exact values whenever suitable.*
☐	☒	For Bayesian analysis, information on the choice of priors and Markov chain Monte Carlo settings
☐	☒	For hierarchical and complex designs, identification of the appropriate level for tests and full reporting of outcomes
☐	☒	Estimates of effect sizes (e.g. Cohen's *d*, Pearson's *r*), indicating how they were calculated

Our web collection on statistics for biologists contains articles on many of the points above.

Software and code

Policy information about availability of computer code

Data collection	Image data was collected with LSM 710 and the software, ZEN 2.
Data analysis	Semi-quantitative analysis of the fluorescence signals was performed using ImageJ 2X or ZEN 2. Quantification of Western blot was performed by ImageJ2x. Statistics were calculated by Microsoft Excel 2019 and GraphPad Prism 8. RNA-seq data are analysed by Cufflinks (v2.2.1). GO analyses were performed using the open gene enrichment analysis tool Metascape (http://metascape.org/).

For manuscripts utilizing custom algorithms or software that are central to the research but not yet described in published literature, software must be made available to editors and reviewers. We strongly encourage code deposition in a community repository (e.g. GitHub). See the Nature Portfolio guidelines for submitting code & software for further information.

Data

Policy information about availability of data

All manuscripts must include a data availability statement. This statement should provide the following information, where applicable:

- Accession codes, unique identifiers, or web links for publicly available datasets
- A description of any restrictions on data availability
- For clinical datasets or third party data, please ensure that the statement adheres to our policy

The single-cell RNA-seq raw data generated in the manuscript is available in the Sequence Read Archive (SRA) database under the accession code PRJNA781008 (https://www.ncbi.nlm.nih.gov/bioproject/PRJNA781008). Source data are provided with this paper.

Human research participants

Policy information about studies involving human research participants and Sex and Gender in Research.

Reporting on sex and gender	*Use the terms sex (biological attribute) and gender (shaped by social and cultural circumstances) carefully in order to avoid confusing both terms. Indicate if findings apply to only one sex or gender; describe whether sex and gender were considered in study design whether sex and/or gender was determined based on self-reporting or assigned and methods used. Provide in the source data disaggregated sex and gender data where this information has been collected, and consent has been obtained for sharing of individual-level data; provide overall numbers in this Reporting Summary. Please state if this information has not been collected. Report sex- and gender-based analyses where performed, justify reasons for lack of sex- and gender-based analysis.*
Population characteristics	*Describe the covariate-relevant population characteristics of the human research participants (e.g. age, genotypic information, past and current diagnosis and treatment categories). If you filled out the behavioural & social sciences study design questions and have nothing to add here, write "See above."*
Recruitment	*Describe how participants were recruited. Outline any potential self-selection bias or other biases that may be present and how these are likely to impact results.*
Ethics oversight	*Identify the organization(s) that approved the study protocol.*

Note that full information on the approval of the study protocol must also be provided in the manuscript.

Field-specific reporting

Please select the one below that is the best fit for your research. If you are not sure, read the appropriate sections before making your selection.

☒ Life sciences ☐ Behavioural & social sciences ☐ Ecological, evolutionary & environmental sciences

For a reference copy of the document with all sections, see nature.com/documents/nr-reporting-summary-flat.pdf

Life sciences study design

All studies must disclose on these points even when the disclosure is negative.

Sample size	No specific statistical methods were used to predetermine the sample size. According to the experience in our previously published studies (Sha QQ et al, Nature Communications 2020; Sha QQ et al, Nucleic Acids Res 2021) to choose an adequate pool for microscopy, qRT-PCR, and mouse experiments. For each experiment, the samples used were indicated in the figures or legends.
Data exclusions	No data were excluded.
Replication	All results were reproduced at least twice.
Randomization	Samples/organisms/participants of the same genotype/age were randomly allocated into the siRNA interfering or control groups.
Blinding	The investigators were blinded to group allocation during oocyte manipulation and RNA-seq data collection and analysis.

Reporting for specific materials, systems and methods

We require information from authors about some types of materials, experimental systems and methods used in many studies. Here, indicate whether each material, system or method listed is relevant to your study. If you are not sure if a list item applies to your research, read the appropriate section before selecting a response.

Materials & experimental systems

n/a	Involved in the study	
☐	☒	Antibodies
☐	☒	Eukaryotic cell lines
☒	☐	Palaeontology and archaeology
☐	☒	Animals and other organisms
☒	☐	Clinical data
☒	☐	Dual use research of concern

Methods

n/a	Involved in the study	
☒	☐	ChIP-seq
☒	☐	Flow cytometry
☒	☐	MRI-based neuroimaging

Antibodies

Antibodies used	The detailed information of primary antibodies is described in Supplementary Table 1.
Validation	All primary antibodies are validated for their specificities. The secondary antibodies with minimal cross-reactivities were purchased from Thermo Fisher Scientific.

Eukaryotic cell lines

Policy information about cell lines and Sex and Gender in Research

Cell line source(s)	HeLa cell is from American type culture collection (ATCC, USA).
Authentication	HeLa cells (ATCC, USA) were authenticated by FuHeng Biology (China).DNA was extracted and amplified with the 20-STR amplification scheme. The STR-locus and Amelogenin were detected on DNA Analyzer (ABI 3730XL).
Mycoplasma contamination	HeLa cells (ATCC, USA) were authenticated and tested for mycoplasma contamination.
Commonly misidentified lines (See ICLAC register)	*Name any commonly misidentified cell lines used in the study and provide a rationale for their use.*

Animals and other research organisms

Policy information about studies involving animals; ARRIVE guidelines recommended for reporting animal research, and Sex and Gender in Research

Laboratory animals	The 4-weeks old C57BL/6 strain female mice were used in this study. Mice were maintained under specific pathogen free conditions in a controlled environment of 20–22℃, with a 12/12 h light/dark cycle, 50–70% humidity, and food and water provided.
Wild animals	No wild animals used in this study
Reporting on sex	The study focus on early embryo development. So, the experimental subjects must be collected from the oviduct of female mice. The sex of early embryos was random and has no effect on the experimental results.
Field-collected samples	This study did not contain samples derived from animals from the field.
Ethics oversight	All experimental protocols involving mice were approved by the Zhejiang University Institutional Animal Care and Research Committee (Approval # ZJU20170014).

Note that full information on the approval of the study protocol must also be provided in the manuscript.
